# Intervention for Diabetes with Education, Advancement and Support (IDEAS) study: protocol for a cluster randomised controlled trial

**DOI:** 10.1186/s12913-016-1782-y

**Published:** 2016-09-29

**Authors:** Jun Yang Lee, Carina Ka Yee Chan, Siew Siang Chua, Chirk Jenn Ng, Thomas Paraidathathu, Kenneth Kwing-Chin Lee, Shaun Wen Huey Lee

**Affiliations:** 1 School of Pharmacy, Monash University Malaysia, Jalan Lagoon Selatan, Bandar Sunway, 47500 Subang Jaya, Selangor Malaysia; 2 School of Psychology, Australian Catholic University, 1100 Nudgee Road, Banyo, QLD 4104 Australia; 3 Jeffrey Cheah School of Medicine and Health Sciences, Faculty of Medicine, University of Malaya, 50603 Kuala Lumpur, Malaysia; 4 Department of Primary Care Medicine, Faculty of Medicine, University Malaya, 50603 Kuala Lumpur, Malaysia; 5 School of Pharmacy, Taylor’s University Malaysia, No. 1 Jalan Taylor’s, 47500 Subang Jaya, Selangor Darul Ehsan Malaysia

**Keywords:** Diabetes management, Remote monitoring, Telemonitoring, Type 2 diabetes mellitus

## Abstract

**Background:**

The high market penetration of mobile phones has triggered an opportunity to combine mobile technology with health care to overcome challenges in today’s health care setting. Although Malaysia has a high Internet and mobile penetration rate, evaluations of the efficacy of incorporating this technology in diabetes care is not common. We report the development of a telemonitoring coaching system, using the United Kingdom (UK) Medical Research Council (MRC) framework, for patients with type 2 diabetes mellitus.

**Methods:**

The Intervention for Diabetes with Education, Technological Advancement and Support (IDEAS) study is a telemonitoring programme based on an empowerment philosophy to enable participants to be responsible for their own health decision and behaviour. An iterative cycle of development, piloting, and collating qualitative and quantitative data will be used to inform and refine the intervention. To increase compliance, the intervention will be designed to encourage self-management using simple, non-technical knowledge. The primary outcomes will be HbA1c, blood pressure, total cholesterol, and quality of life and diabetes self-efficacy. In addition, an economic analysis on health service utilisation will be collected.

**Discussion:**

The mixed-method approach in this study will allow for a holistic overview of using telemonitoring in diabetes care. This design enables researchers to understand the effectiveness of telemonitoring as well as provide insights towards the receptiveness of incorporating information technology amongst type 2 diabetes patients in a community setting.

**Trial registration:**

ClinicalTrials.gov NCT02466880 Registered 2 June 2015.

## Background

Globally, an estimated 385 million people are living with diabetes, and this is expected to increase to 552 million by 2030 [1, 2] . This increasing prevalence of diabetes, especially type 2, poses a major public health concern, as it is associated with substantial morbidity, mortality, as well as financial cost due to the associated microvascular and macrovascular complications. In Malaysia, type 2 diabetes affects approximately 12 % of the total population and, together with ischaemic heart disease, constitute nearly 75 % of the total non-communicable disease burden [3]. Clinical care of diabetes currently consumes approximately 16 % of the total national healthcare budget in Malaysia, with an estimated RM2.4 billion (about USD0.55 billion) spent in 2010 [4]. A recent study in Malaysia reported that patients with diabetes spent up to 14.7 % of their household income on outpatient diabetes care [5].

The utilization of self-monitoring blood glucose (SMBG) for individuals with diabetes is low, with only 6.9 and 26.8 % of people with diabetes utilising SMBG in private and public hospitals, respectively [6]. The utilisation of SMBG in people with diabetes is complex. Financial, psychological, and behavioural factors can affect an individual’s utilisation of SMBG [6–8]. Innovative methods are required to overcome these challenges. Currently, with the advancement of technology, there is a promising potential to overcome such barriers [9, 10].

Remote patient monitoring or telemonitoring has recently gained much attention as a promising and innovative solution to improve patient care and management from a distance, especially for those with diabetes. It involves the use of telecommunication technologies such as video, audio, handheld technologies or intelligent sensors for the digital transmission of physiological and other disease related data for monitoring patient’s condition and clinician’s feedback [11, 12]. Systematic reviews of telemonitoring intervention in diabetes, asthma, heart failure and hypertension showed that telemonitoring is a safe, effective and acceptable intervention method with better glycaemic control, significant improvements in peak respiratory flows and reduction in systolic or diastolic blood pressure [13, 14]. Additionally, due to the algorithmic structure of computerised systems, data reported by patients were entirely accurate, overcoming the challenges of inaccurate results [15–17].

However, there is limited evidence regarding the feasibility of translating telemonitoring research into practice in a developing country such as Malaysia. Malaysia with a population of 29 million people has a mobile penetration rate of 140 %, indicating that 47 % of Malaysians own more than one mobile phone [18, 19]. Furthermore, Malaysia has a total of 20 million Internet users and 15 million people are subscribers of third generation (3G) networks, indicating a potential to incorporate mobile telemonitoring among the general public [18, 19]. Therefore, this study will design a system, which incorporates current technology, and evaluate the practicality of such a system in patient care, for people diagnosed with type 2 diabetes in Malaysia. The focus of the study will be on the use of remote monitoring to maximise the use of information and communications technology (ICT) to deliver lifestyle intervention, provide accurate data, as well as influence patients’ attitude and behaviour in a community setting in Malaysia. This study will include various factors working interdependently with each other, including an automated feedback mechanism for diabetes monitoring, cost effectiveness, diabetes education as well as a focus group to understand participants point of view for future intervention implementation. The UK Medical Research Council (MRC) framework for complex intervention will be used to guide the development and evaluation of this current study.

**Fig. 1:**
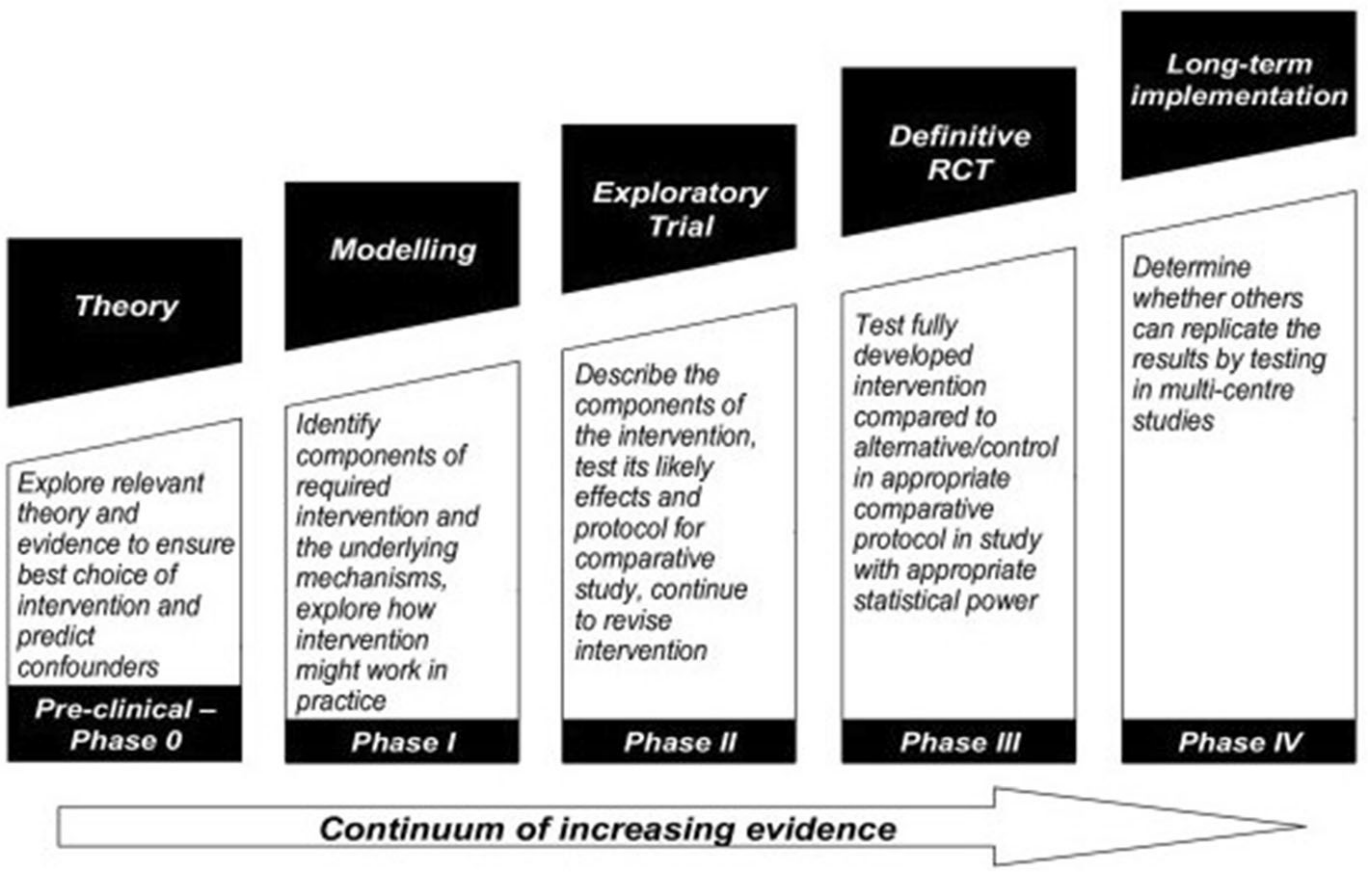
MRC framework adapted from Higginson et al. *BMC Palliative Care* 2006 5:7 [22]

This framework emphasises that the development, evaluation and implementation of any complex intervention require a strong theoretical foundation [20, 21]. Furthermore, a wide spectrum of intervention studies which required behavioural changes, information services, education programmes, integrated systems of patient care and complementary therapies have been conducted using this approach [21, 22]. The MRC framework suggests a progression of different phases, starting from the pre-clinical phase up to phase IV clinical trials (Fig. 1). This article will discuss how the MRC framework criteria are used to develop the current study.

## Methods/design

### Aim of the study

The aim of this cluster randomised controlled study is to evaluate the effectiveness of the Intervention for Diabetes with Education, Technological Advancement and Support (IDEAS) among type 2 diabetes adults, in reducing serum HbA1c levels. The IDEAS involves data transmission, feedback and education.

### Outcome measures

The primary outcome of this study is changes in HbA1c levels pre and post intervention. Secondary outcome measures include changes in body weight, fasting blood glucose, blood pressure, quality of life and physical activity.

### Theoretical framework

The Health Belief Model (HBM) forms the basis for the theoretical underpinning of this study [23]. HBM is a conceptual framework that has been used extensively to explain health behaviour [24]. However, the theoretical HBM does not suggest a strategy for changing health-related actions. Therefore, modifying factors which include media, health professionals, personal relationships, incentives and self-efficacy of recommended health action create a “cue to action” for patients to elicit behaviour compliance.

### Articulation of study to HBM framework

Perceived barriers to self-care can hinder progress in patient participation. Several perceived barriers deter patient participation in regular SMBG. These include inconvenient programme days and time, lack of feedback, lack of reminders and the cost [25]. This study is designed to address these common barriers. To make participation convenient, follow-up sessions will be scheduled during participants’ regular appointments at the clinics. To address the barrier of time, diabetes classes will last for a maximum of one hour, screening and follow-up consultations will take approximately 1 h, while focus group sessions will last up to 2 h, will be conducted at separate sessions. Therefore, total contact time commitment for the entire programme will be seven and a half hours over a span of 1 year. To overcome the lack of feedback, participants in the telemonitoring group will receive an automated e-mail reminder if self-monitoring of blood glucose is not performed for three consecutive times while monthly follow-up calls to participants in the control group will be conducted. Furthermore, feedback will be provided to participants if blood glucose results show three continuous blood glucose values of ≤ 3.9 mmol/l or ≥11.1 mmol/l in the telemonitoring group. To overcome the cost barrier, the programme will be offered free to all participants.

### Multidisciplinary committee

In order to enhance the community-specific intervention of the study, a multidisciplinary committee will be established to receive input from various experts including doctors, pharmacists and nurses. The committee will also provide assistance in participant enrolment, patient counselling and disease education.

### Phase 0–1: theoretical phase/modelling

The theoretical basis of this study was established by reviewing published literature on the utilisation of mobile technology in diabetes care [26–31]. To develop a cultural-specific telemonitoring system that is suitable for the Malaysian population, evidence of poor diabetes management and its additional associated risks during Ramadan was reviewed [32, 33]. This allowed for specification of the required interventions that would accommodate an ethnically diverse country like Malaysia.

### Phase 2: exploratory trial

This exploratory phase of the study focused on the use of telemonitoring during the holy month of Ramadan. During this period, Muslims are required to refrain from consuming food and water from dawn till dusk. Studies have shown that modified dietary habits could increase the risk of severe hypoglycaemia by nearly 7.5-fold [33, 34]. Therefore, the objective of this exploratory phase was to evaluate the effects of implementing a telemonitoring programme to aid Muslims with type 2 diabetes who were fasting during Ramadan. Ethics approval for this study has been obtained from the Medical Research and Ethics committee, Malaysia (NMRR-14-177-19466) and Monash University Research Ethics Committee (CF14/1977-2014001016).

Eligible participants included those who had been diagnosed with type 2 diabetes, aged 18–75 years, with HbA_1c_ level between 7.5 and 11 %, willing to fast for 15 days, had internet access, an e-mail address and a smartphone, and provided informed written consent. Patients from five public health care centres were allocated into usual care group (*n* = 19) *or* telemonitoring group (*n* = 18) using clustered randomisation method. Education materials which focused on diabetes management during Ramadan were provided to participants to standardise education level as well as diabetes management practice.

**Fig. 2:**
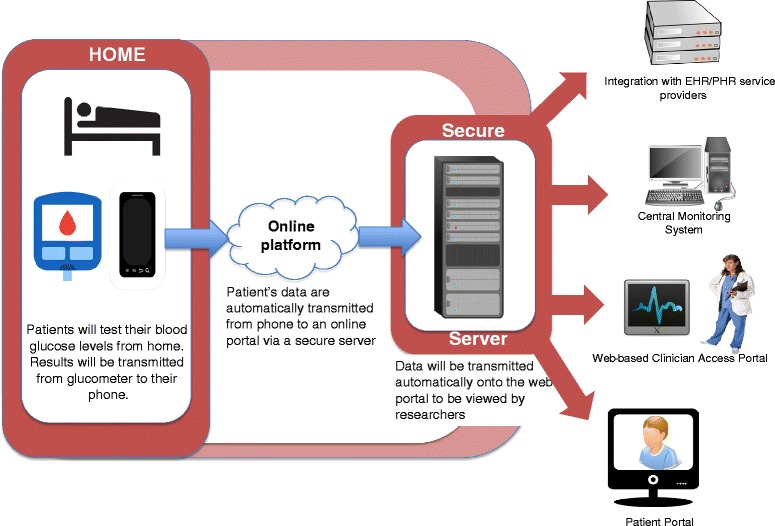
Technical schema of the proposed telemonitoring system

During the 7-week intervention, a web-enabled glucose meter (Entra Health System, San Diego, CA, USA), connected to an android phone, which could transmit recorded blood glucose results to a web portal, was provided to all participants in the telemonitoring group. The device also provided automatic feedback to the participant if his/her recorded blood glucose results for three continuous tests were ≤ 3.9 mmol/l or ≥11.1 mmol/l. A schematic diagram representing the elements of this system is shown in Fig. 2. Diaries were provided to all participants in the study to record any hypoglycaemic events that occurred during the study. Participants were encouraged to self-monitor their blood glucose levels five times a day during the month of Ramadan. Ten focus group sessions were conducted to elicit participants’ experiences of fasting during the month of Ramadan as well as the study design.

Results from the pilot study showed that hypoglycaemic episodes were lower in the telemonitoring group compared to the usual care group [35]. Focus group discussion indicated that participants were open to new technologies, which helped them in managing their disease. Several limitations were noted during the exploratory phase of the study. These were mostly related to the study design. Since participants had to be recruited when they were fasting during the month of Ramadan, this limited the recruitment time and hence, the small sample size. In addition, difficulty in accessing Internet connection affected the delivery of the blood glucose results to the researchers.

### Randomised controlled trial (phase III)

The exploratory phase was conducted in a specific population within a seasonal time period and generalisability of the results could be limited. Generalisation to a larger and more diverse population remains to be tested. To overcome these limitations, we describe the design of a larger study (IDEAS Study), which will take into considerations the limitations noted in the pilot study.

### Randomisation

The study will follow a cluster randomised design with the public clinics as the unit of randomisation. This will avoid potential contamination between individuals who are assigned to different treatment groups in the same study site. A trained individual, independent of the study team will conduct all the randomisation.

### Sample size calculation

A previous study in a tertiary hospital reported a 0.79 % mean difference in HbA1c between adherent and non-adherent people with diabetes [36]. A sample size of 100 people per study arm will have an 80 % power to detect a 0.5 % difference in HbA1c between the intervention and control arm. Therefore, after accounting for a 20 % loss to follow-up in each arm, 240 people with diabetes will be recruited in this study.

### Sample recruitment procedures

A range of recruitment strategies will be used to recruit participants. Investigators will work closely with the doctors as well as the nurses at the study sites to promote and identify participants diagnosed with type 2 diabetes. Participants may also be self-referred or referred through their primary care doctors.

Selection criteria include people: (1) with type 2 diabetes based on diagnosis by a doctor, for at least 6 months prior to study enrolment, determined via self-report with verification (medical records, current treatment); (2) aged 18–75 years; (3) residing in the state of Selangor without plans to leave for the next 12 months; (4) with HbA1c levels of ≥ 7.5 % but less than 11.0 % within the most recent 3 months; (5) who have regular access to internet and an e-mail address or access to a smartphone with 3G services. Participants will be excluded if they: (1) are unable or unwilling to give written informed consent or communicate with local study staff; (2) are currently diagnosed with mental disorder; (3) have been hospitalised for depression in the past six months; (4) do not have support from primary healthcare provider or family members; (5) are known to have history of bariatric surgery, small bowel resection, or extensive bowel resection, and (6) are currently pregnant or breastfeeding.

### Control arm

Participants in the control arm will be advised to continue their routine healthcare with their doctors, including self-management of blood glucose and recording of results in log books.

### Intervention arm

**Fig. 3:**
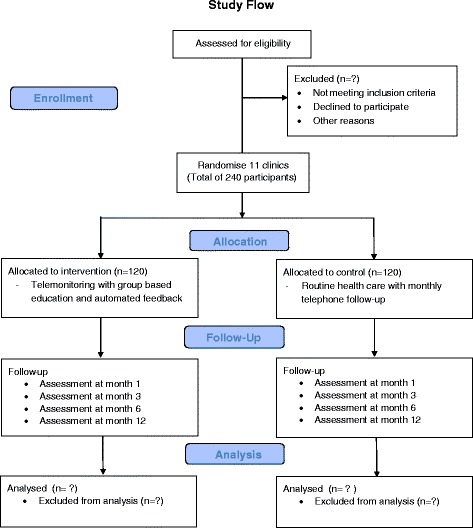
Schematic diagram of study trial process

Participants in this arm will be encourage to self-monitor their blood glucose levels and will be provided with a group-based diabetes education, an automated feedback mechanism to both patient and family member(s) as well as an electronic logbook. The web-enabled function of the web-based glucose meter will be utilised, which when connected to an android enabled smartphone device, will automatically upload any blood glucose readings to an online portal which is currently managed by the Entra Health System. The web portal will host participant’s glycaemic and metabolic results as well as other information such as self-management skills, compliance to eye measurements and screening for microvascular complications. The system will be calibrated to generate a message to inform the attending doctor or researcher in the event that three consecutive readings of hypoglycaemia (3.9 mmol/L or below) or hyperglycaemia (11.1 mmol/L and above) are detected. A schematic diagram representing the elements of this system is shown in Fig. 2. The doctor or researcher will have the option to use this information to initiate an intervention. The doctor will be responsible for all treatment decisions. Participants in the intervention arm will receive monthly communications from the research team. Communications will focus on self-management skills, blood glucose control, and medication adherence. Figure 3 shows the process of the study trial.

### Focus group

To better understand participants’ perception towards this research, focus group sessions will be conducted. Interested participants will be given an overview of the purpose of the focus group discussion to ascertain their interest in participating in this discussion. These sessions will last between one and a half to two hours, and each session will consist of not more than eight participants, who will engage in discussion on the topics under consideration. Selection of participants will be based on common characteristics among them. A semi-structured interview protocol using open-ended questions to encourage group discussion, will be used and a trained moderator will conduct the focus group sessions. The moderator will provide nondirective, supportive and non-evaluative role. He/she is also responsible to facilitate participants’ responses and to prevent deviation from the topics being discussed. It is estimated that a total of five focus groups will be required to achieve saturation. Focus group sessions will be held at the times convenient to the participants. Interviews with the various healthcare professionals, including doctors and nurses, will be conducted to understand their opinion and acceptability in adapting telemonitoring into current Malaysian healthcare settings.

### Measurements

Table 1 details the measures that will be assessed throughout the study. HbA1c will be analysed using venous samples by ion-exchange high performance liquid chromatography (Cobas Integra 800, Roche), and serum lipids using enzymatic techniques (Advia 2400, Siemens). All blood specimens will be collected and analysed in a central laboratory operated by Gribbles Pathology, Malaysia to reduce variability.

**Table 1:** Primary and secondary outcome measures for visit 0, 4, 12, 24, 52

Variable	Instrument
Primary outcome variable	
Glycaemic control	HbA1c
Secondary outcome variable	
Glycaemic control	Fasting plasma glucose
Blood lipids	Total cholesterol, LDL cholesterol, HDL cholesterol, triglycerides
Kidney function	Creatinine
Blood pressure	Measured twice using A&D UA-767 Plus BT-C blood pressure device on the same arm.
Blood oxygen saturation and pulse rate	Spo2; using Nonin Oximeter II Model 9560 on the right index finger.
Body Mass Index (BMI)	Calculated from height and weight measured by research staff.
Self-efficacy	Self-efficacy scale [46]
Diabetes distress	Diabetes distress scale [37]
Diabetes self-care	Diabetes self-care activities questionnaire [38]
Quality of life	EQ-5D [40]
Healthcare utilization	Self-reported using healthcare utilization questionnaire [39]
Smoking	Self-reported
Co-morbidities	Self-reported

*LDL* low density lipoprotein, *HDL* high density lipoprotein

Participants’ blood pressure will be calculated using the mean of two measurements performed with participants seated after at least 10 min rest, with the cuff on the predominant arm at the level of the heart (A&D, UA-767, USA). Body height and weight will be measured in light indoor clothing and without shoes using a stadiometer and a scale (A&D, UC-321 PBT-C, USA), respectively. Blood oxygen saturation and pulse rate will be measured using an oximeter (Nonin Oximeter II, Model 9560, USA).

Several generic and disease-specific instruments will be used in the current study to measure participants’ diabetes knowledge, quality of life, self-efficacy, diabetes distress and health care utilization. These include the diabetes distress scale [37], diabetes self-care activities [38], health care utilization [39] and EuroQol-5D (EQ-5D) [40]. Questionnaires will also be translated to Bahasa Malaysia and Mandarin if required, and subsequently validated.

### Data analysis

#### Data quality

A unique study identifier will be used to tag all study forms and biological samples which are collected during this study. Quantitative data will be entered into a database and audited for accuracy. In order to maintain participant confidentiality, coded form will be kept separately from the code list. Any missing and outlier values will be screened by a research assistant and hence, extensive training of research assistants is important to ensure quality of data entry.

#### Quantitative data analysis

Analysis of quantitative study data across randomised groups will be conducted in IBM SPSS version 20 (Armonk, NY). Continuous variables will be tested for normality and data, which are not normally distributed, will be analysed using Kruskal-Wallis and Wilcoxon signed ranks test. In the event of missing data, the last observation carry forward method will be used. A *p* value of < 0.05 will be considered as statistically significant for all the tests.

Effectiveness of the research intervention will be measured in terms of differences between study arms with respect to changes in biomedical measures and activities. Logistic regression will be used and both adjusted and unadjusted models will be presented. Benefits, barriers, confidence and knowledge will be included in a model to identify factors associated with the concept of intensive treatment and hypoglycaemia. An adjusted *t*-test using multiple linear regression analysis will be conducted to calculate variables for benefits, barriers and knowledge.

#### Qualitative data analysis

Focus group discussion will be transcribed verbatim and qualitative responses will be independently reviewed by the researchers (one psychologist, one pharmacist and one research assistant) to identify themes and develop a coding scheme.

### Cost-effective analysis

We will conduct a literature review to identify studies, which critically assessed the cost-effectiveness of an intervention in diabetes in this region. We will also collect data to examine healthcare resource use and indirect costs using standard economic analytical tools. Cost analysis using direct and indirect costs will be performed on the two study groups based on the number of referrals to specialists (e.g. endocrinologists, optometrists, podiatrist, dietitians), total clinic attendance and prescribed medications and tests linked to diabetes. The indirect costs will include travel cost, and cost such as days of sick leave from work and premature early retirement due to diabetes. The impact on quality of life will be assessed using EQ-5D scale by interviewing patients from the two groups before and after the intervention. This data will be used to estimate the cost/Quality Adjusted Life-Year (QALY) in cost utility analysis.

## Discussion

The IDEAS study is designed to assess the feasibility, effectiveness and cost effectiveness of a telemonitoring programme for type 2 diabetes patients in primary care settings. Previous studies have shown that telemonitoring is effective to improve health behaviour and encourage self-management of chronic diseases [13, 41, 42]. However, the management of diabetes remains a complex issue involving various aspects which include behavioural, financial, and psychological factors. The inclusion of various support structures especially from family members and doctors have produced positive results but further studies are required to incorporate these findings into a real-life community setting in a developing country, such as Malaysia [43–45].

This mix-method study that incorporates both quantitative and qualitative methods will allow better understanding of the effectiveness as well as the receptiveness of telemonitoring in a Malaysian community setting. Additionally, insights from focus group discussion in this study ensure that the intervention is relevant to a multi-ethnic and culturally diverse country like Malaysia. Therefore, this study will provide valuable information in evaluating the effectiveness, acceptability and cost-effectiveness of incorporating telemonitoring system in managing diabetes in the Malaysian health care system. Possible important limitations can be found in this study. Due to the online nature of this study, results of SMBG transmitted online may be delayed in areas with limited internet access. Additionally, participants who are less technology savvy will require more education in using the new technology.

Recruitment of participants commenced in April 2015, and data collection is scheduled to complete by December 2016, after which final data analysis will be performed and the findings will be presented in a separate publication.

### Relevance to public health

The results of this study will be of relevance to policy makers on the importance of diabetes management using telemonitoring. The 6-month follow-up period will provide valuable data on the sustainability of the effect of such programme over time. The findings of this study will also enable better approaches to health promotion and the management of chronic diseases and their complications as well as serve as a source of information on the success and limitations that entailed in the implementation of a telemonitoring programme.

## Abbreviations

3G: Third generation
CEA: Cost-effectiveness analysis
CUA: Cost-utilities analysis
EQ-5D: EuroQol-5D
HBM: Health belief model
HDL: High density lipoprotein
ICT: Information and communications technology
IDEAS: Intervention for Diabetes with Education, Technological Advancement and Support
LDL: Low density lipoprotein
MRC: Medical Research Council
SMBG: Self-monitoring blood glucose
UK: United Kingdom
